# Supplementary material for: Interleukin-8 and Interleukin-6 Are Biomarkers of Poor Prognosis in Esophageal Squamous Cell Carcinoma
Source: Cancers (Basel). 2023 Mar 27;15(7):1997. doi: 10.3390/cancers15071997 (PMC10093339; doi:10.3390/cancers15071997)

Table S1 – Calculation of power and effect size for the realization of the Cox regression model.

Variable of interest	SD	R2	HR	Power
**IL-12**	0.461	0.190	0.326	0.94
**TNF-α**	0.468	0.051	0.226	> 0.99
**IL=8**	0.468	0.218	4.564	> 0.99

IL: Interleukin; TNF-α: Tumor necrosis factor α; SD: standard deviation of covariate of interest; R2: squared coefficient of multiple correlation with other covariates; HR: hazard ratio (exponentiated b1) associated in covariate of interest.
For this calculation, the software Stata v.14.0 was used under the powercox function.

Table S2 - Pearson's correlation coefficient

Covariable	r*	p
**Race**	0.073	0.635
**IL-12**	0.276	0.067
**TNF-α**	-0.153	0.315
**IL-8**	-0.01	0.95
**Surgery**	-0.223	0.14
**Radiotherapy**	-0.175	0.251
**Chemotherapy**	0.197	0.194
**TNM**	-0.077	0.614

* Pearson's correlation coefficient

Table S3 – Characterization of the study population in relation to socio-demographic and lifestyle variables.

Variable / Category	Cases		Controls		P
	n(*)	(%)	n (*)	(%)	
**Sex**					
Male	59	84.3	58	82.9	0.820
Female	11	15.7	12	17.1	
**Race**					
White	50	73.5	51	72.9	0.929
Non-white	18	26.5	19	27.1	
**Marital status**					
Not married	17	24.3	22	31.4	0.346
Married	53	75.7	48	68.6	
**Degree of education**					
Illiterate / functional illiterate	10	14.3	0	0.0	**<0.001**
Elementary and middle school	56	80.0	54	77.1	
Higher education	4	5.7	16	22.9	
**Place residence**					
Urban area only	12	17.1	29	41.4	**0.007**
Rural area only	8	11.4	5	7.1	
Both areas	50	71.4	36	51.4	
**Alcohol**					
Never	3	4.3	12	17.1	**<0.001**
Yes, currently (if it was stopped in the last 12 months)	36	51.4	49	70.0	
Yes, in the past	31	44.3	9	12.9	
**Tobacco**					
Never	10	14.3	37	52.9	**<0.001**
Yes, currently (if it was stopped in the last 12 months)	47	67.1	11	15.7	
Yes, in the past	13	18.6	22	31.4	
**Exposure to pesticide or insecticide**					
No	28	40.0	50	73.5	**<0.001**
Yes	42	60.0	18	26.5	
**Practice physical activity**					
No	49	70.0	36	51.4	**0.024**
Yes	21	30.0	34	48.6	

Utilized test: Chi-square
Statistically significant if p <0.05

Table S4 – Characterization of the case study group in relation to clinicopathological variables.

Variable	Category	n (*)	(%)
**Topography of the tumor**	Upper third	2	(3.7)
	Middle third	31	(57.4)
	Lower third	2	(3.4)
	SOE	19	(35.2)
**Degree of differentiation**	Well differentiated	9	(13.8)
	Moderately differentiated	35	(53.8)
	Little differentiated	21	32.3)
**T**	T1 – T2	11	(17.5)
	T3 – T4	52	(85.5)
**N**	N0	19	(34.5)
	N positive	36	(65.5)
**M**	M0	56	(84.8)
	M1	10	(15.2)
**TNM staging**	I – II	17	(28.8)
	III – IV	42	(71.2)
**Total**		**70**	**(100)**

(*)Cases with ignored values were excluded from the analysis.
SOE: Tumor located in more than one region of the oesophagus.
TNM staging: System based on the size and / or extent of the primary tumor (T), amount of compromised lymph nodes (N) and presence of metastases (M).

Table S5 – Cutoff for cytokines.

Cytokines	Cutoff	Sensitivity (%)	Specificity (%)	AUC (%)
**IL-12p70**	1.23	75.6	37.5	59.3
**TNF-α**	0.825	75.6	42.0	57.7
**IL-10**	1.34	75.6	33.3	49.5
**IL-1β**	1.35	77.8	30.0	55.8
**IL-6**	4.7	77.8	71.0	76.0
**IL-8**	6.75	73.3	71.0	76.0

AUC: Area under the curve ROC; IL: Interleukin; TNF-α: Tumor necrosis factor α.
Utilized test: ROC curve to find a cutoff point of cytokine levels and then dichotomize at low and high levels.

Table S6 – Descriptive of the categorized cytokines.

Cytokines	Categories (level)	n (%)
**IL-12p70**	Low (<1.23pg/mL)<br>High (≥1.23 pg/mL)	49 (70.0)<br>21 (30.0)
**TNF-α**	Low (< 0.825 pg/mL)<br>High (≥ 0.825 pg/mL)	48 (68.6)<br>22 (31.4)
**IL-10**	Low (< 1.34 pg/mL)<br>High (≥ 1.34 pg/mL)	50 (71.4)<br>20 (28.6)
**IL-1β**	Low (< 1.35 pg/mL)<br>High (≥ 1.35 pg/mL)	52 (74.3)<br>18 (25.7)
**IL-6**	Low (< 4.7 pg/mL)<br>High (≥ 4.7 pg/mL)	27 (38.6)<br>43 (61.4)
**IL-8**	Low (< 6.75 pg/mL)<br>High (≥ 6.75 pg/mL)	29 (41.4)<br>41 (58.6)

n: number; IL: Interleukin; TNF-α: Tumor necrosis factor α; pg/mL: picogram per millilitre.

Table S7 - Relative expression of mRNA in tumor tissues induced by Kyse-30 and Kyse-410 cell lines.

Gene	Kyse-30 tumors	Kyse-410 tumors	p value
CXCL1	2163 ± 455.1 (n=9)	4775 ± 1380 (n=6)	**0.0253**
VEGF	62759 ± 7422 (n=5)	36033 ± 1512 (n=3)	0.3938
CCL2	1631 ± 247.1 (n=5)	547.5 ± 64.52 (n=3)	0.0794
CXCL2	Not detected	Not detected	

Quantitation of the mRNA levels of the genes by qPCR were calculated using the 2-ΔCT method. ΔCT was calculated by subtracting the CT (control: ubiquitin) from the CT (target gene).

Figure S1 – Flowchart of inclusion of patients in the study.

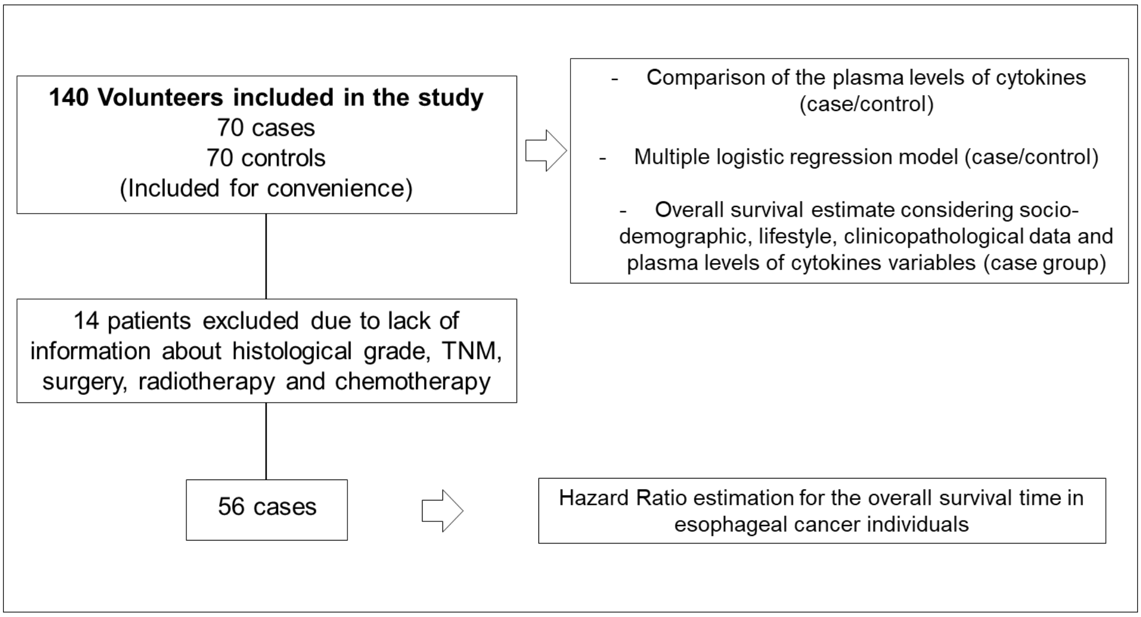

Figure S2 - Kaplan-Meier curve of overall survival of individuals with esophageal cancer (n = 70).

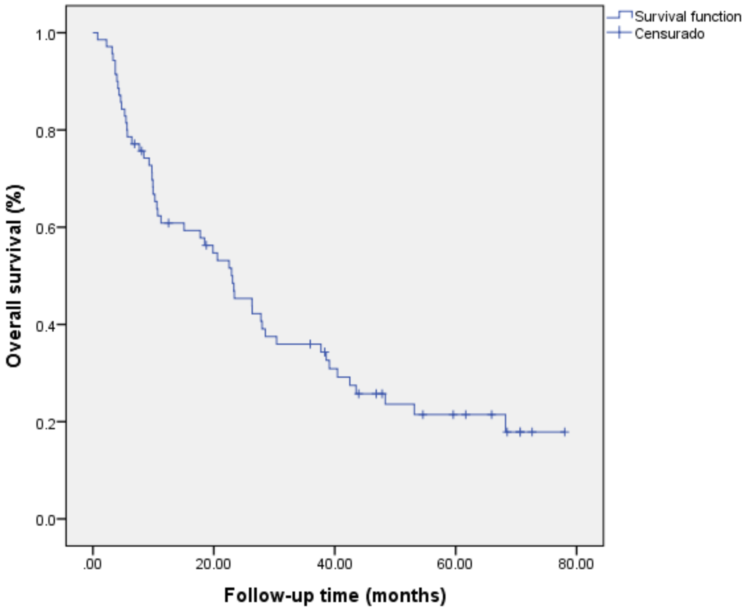

Figure S3 - Kaplan-Meier curves of overall survival correlated with a) IL-6 (n = 70; 43 with high level and 27 with low level), b) IL-8 (n = 70; 41 with high level and 29 with low level), c) TNM (n = 59; 17 with TNM I / II and 42 with TNM III / IV) and d) Surgery (n = 64; 44 did not undergo surgery and 20 underwent surgery).

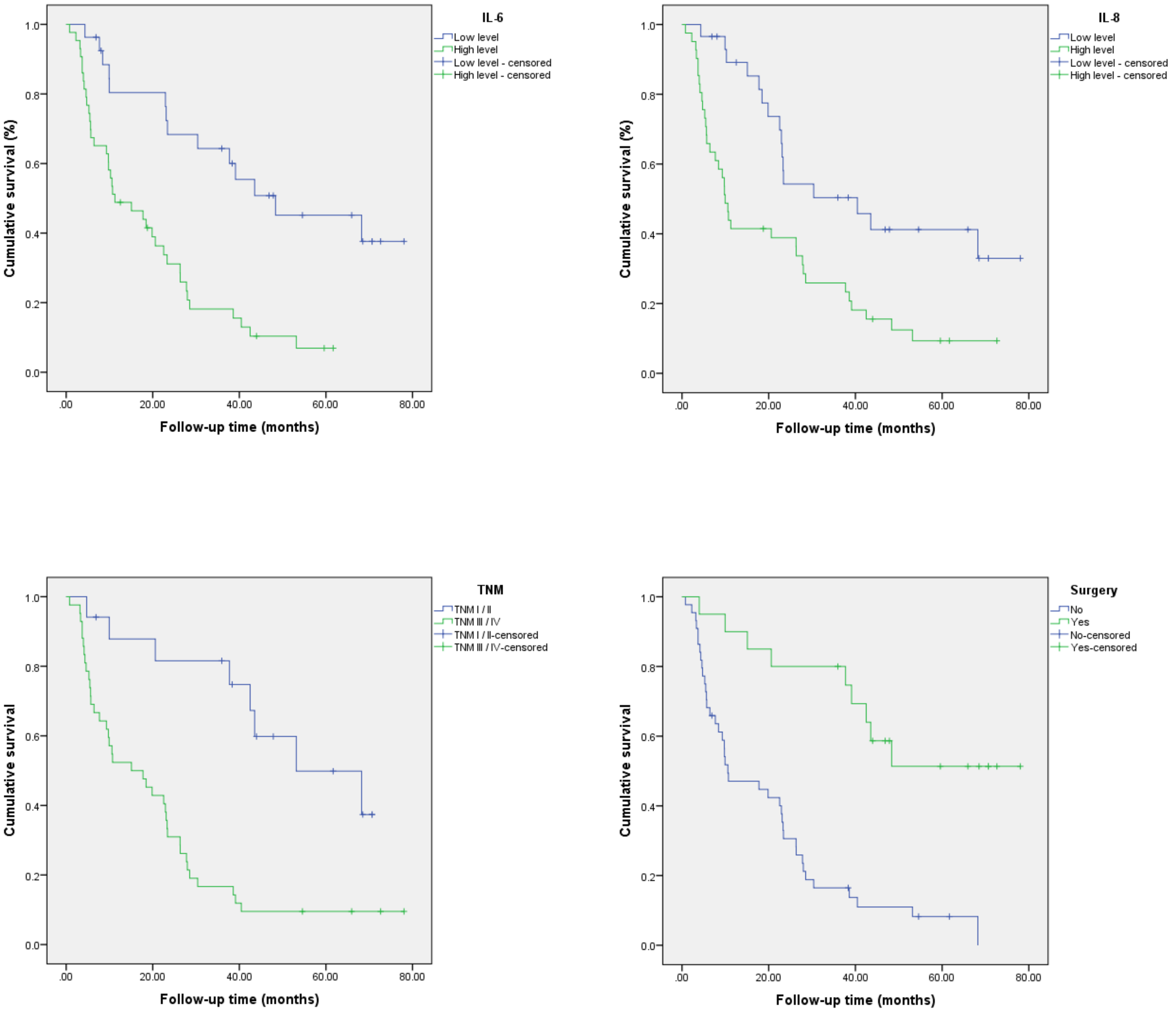

Figure S4 – Gating strategy used for the analysis of intratumoral polymorphonuclear leukocytes (CD11b+ Ly6G+) in Kyse-30 or Kyse-410 tumor-bearing mice.

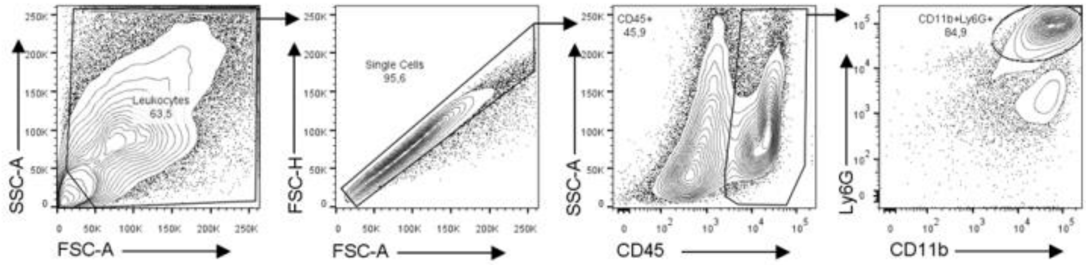

Supplement: Supplementary file 1 [file cancers-15-01997-s001.zip › cancers-2170134-supplementary.pdf]
